# Clinical Predictive Models for Delayed Cerebral Infarction After Ruptured Intracranial Aneurysm Clipping for Patients: A Retrospective Study

**DOI:** 10.3389/fsurg.2022.886237

**Published:** 2022-06-07

**Authors:** Jun Su, Hao Huang, Yuan-jun Xin, Yi-dan Liang, Xin-tong Wu, Xiao-lin Yang, Xiao-zhu Liu, Zhaohui He

**Affiliations:** ^1^ Department of Neurosurgery, The First Affiliated Hospital of Chongqing Medical University, Chongqing, China; ^2^ Department of Neurosurgery, The People’s Hospital of NanChuan, Chongqing, China; ^3^ Department of Neurosurgery, People’s Hospital of Chongqing Hechuan, Chongqing, China; ^4^ Department of Neurosurgery, Chongqing Emergency Medical Center, Chongqing, China; ^5^ Department of Cardiology, The Second Affiliated Hospital of Chongqing Medical University, Chongqing, China

**Keywords:** clipping, delayed cerebral infarction, risk factors, nomogram, ruptured Intracranial aneurysm

## Abstract

**Objective:**

A nomogram was developed in this work to predict the probability of delayed cerebral infarction (DCI) after ruptured intracranial aneurysms (RIA) clipping.

**Methods:**

Clinical data of patients with intracranial aneurysm were obtained from the neurosurgery department of the First Affiliated Hospital of Chongqing Medical University from January 2016 to December 2020. A total of 419 patients receiving surgery of ruptured intracranial aneurysm clipping were included and a total of 37 patients with DCI were set as the observation group. The control group consisted of 382 patients without DCI. Risk factors of DCI were screened by univariate and multivariate logistic regression analysis and included in the nomogram.

**Results:**

Univariate analysis showed that female (*P* = 0.009), small aneurysm (*P* = 0.031), intraoperative aneurysm rupture (*P* = 0.007) and cerebral vasospasm (*P* < 0.001) were risk factors for postoperative DCI while smoking history (*P* = 0.044) were protective factors for postoperative DCI. Multivariate Logistic regression analysis showed that small aneurysm (*P* = 0.002, OR = 3.332, 95%–7.104), intraoperative aneurysm rupture (*P* = 0.004, OR = 0.122, 95%-CI, 0.029–0.504)and cerebral vasospasm (*P* < 0.001, OR = 0.153, 95%-CI, 0.070–0.333) were independent risk factors of postoperative DCI. The calibration curve of the probability of occurrence showed that the nomogram was in good correspondence with the observed results with a C-index of 0.766 (95% CI, 0.684–0.848). Meanwhile, the Decision curve analysis (DCA) showed that the established predictive model had a good clinical net benefit.

**Conclusion:**

The well-established nomogram is expected to be an effective tool to predict the occurrence of DCI after intracranial ruptured aneurysm and can be used to assist clinicians to develop more effective treatment strategies and improve the prognosis of patients.

## Introduction

Clipping of intracranial aneurysm is a traditional surgical method with widely recognized efficacy and safety. Even though the intracranial aneurysm patients are treated in time, there are nearly 30% of them suffer from serious sequelae and the martality rate is approximately 12.1% (1). Ischemic complications after aneurysm clipping are the main cause of death and disability of patients with intracranial aneurysms (2). Early postoperative cerebral infarction mostly occurs near the ruptured aneurysms, it is mostly related to brain injury caused by ruptured aneurysms and surgical operations during the progress of aneurysm clipping. Delayed cerebral infarction (DCI) refers to new cerebral infarction occurred 72 h to 21 d after intracranial aneurysm clipping, which is fundamentally different from early cerebral infarction and is recognized to be closely related to cerebral vasospasm (3). Some patients present with neurological dysfunctions such as hemiplegia, aphasia, and increased disturbance of consciousness after surgery, and some are asymptomatic, only show new partial low or high density signals on Computed Tomography (CT) scan/diffussion weighted Imaging (DWI) or FLAIR. Patients with neurological dysfunctions may not gain complete recovery, and lesions are always present on imaging (4). The pathological mechanism and risk factors of DCI have not been fully elucidated, it may be related to microthrombosis, diffuse cortical depolarization and neuroinflammation (5) besides to cerebral vasospasm, which deserve our further study.

Early identification and positive treatment to reduce postoperative DCI are of great significance. The constructed nomogram is a simple tool for quantifying risk and provide a more accurate and personalized prediction for patients’ early outcomes (6). However, there was no clinical prognostic model specifically for DCI after RIA clipping in the current study and our work is the first prognostic model specifically focused on DCI, which fills the gap in the field of prognostic judgment.

The purpose of this study is to screen the risk factors for postoperative DCI and construct the prognosis nomogram that could reduce the occurrence of postoperative DCI and improve the prognosis of patients.

## Data and Methods

### General Information

Patients who received IA clipping in the neurosurgery department of the First Affiliated Hospital of Chongqing Medical University from January 2016 to December 2020 were selected as the research subjects. Inclusion criteria: (1) intracranial aneurysm confirmed by Digital Subtraction Angiography (DSA) or Computed Tomography Angiography (CTA); (2) Clipping of intracranial aneurysm; (3) New diagnosed cerebral infarction by computed tomography (CT) or Magnetic Resonance Imaging (MRI) 72 h to 21 d after operation. Exclusion criteria: (1) unruptured intracranial aneurysm; (2) incomplete clinical data; (3) New neurological dysfunction (or aggravation on the original disease) was found within 3 days after surgery and head CT was not reviewed; (4) Patients with serious organ diseases, malignant tumors or hematological diseases. As a result, a total of 419 patients including 137 males and 282 females aged from 28 to 73(53.20 ± 9.61) years were included. Among them 37 patients with DCI were set as the observation group and 382 patients without DCI were set as the control group.

### Methods

Gender, age, history of hypertension, history of diabetes, smoking history, drinking history, Hunt-Hess grade, WFNS (World Federation of Neurosurgeons) grade and Modified Fisher Grades, aneurysm location, size, number and morphology, operation opportunity, operation duration, whether intraoperative aneurysm rupture during the operation, parent artery occlusion, cerebral vasospasm and other clinical data were collected from the patients in the observation group and the control group. In this study, aneurysms with the maximum diameter less than 5 mm were small aneurysms. Modified Fisher grade: 0, no hemorrhage or only intraventricular hemorrhage or parenchymal hemorrhage; Grade I, only basal cisternal hemorrhage; Grade II, only peripheral cistern or lateral cistern hemorrhage; Grade III, extensive subarachnoid hemorrhage with brain parenchymal hematoma; Grade IV, the basal cisterns, peripheral cisterns and lateral fissure cisterns with thicker hematoma. CT scan was performed at 24 h, 48 h, 3 d, 7 d, 14 d, and 21 d after aneurysm clipping, and extra CTA and DWI were performed at 3 d, the CT scan or DSA and DWI were reexamined when it comes to new neurological dysfunctions or heavior symptoms. When the diameter of the cerebral blood vessel was smaller than that before surgery, it was identified as cerebral vasospasm. The new partial low or high density signals on CT scan/DWI or FLAIR were judged as DCI (4). The risk factors of DCI after IA clipping were screened by univariate and multivariate Logistic regression analysis.

64-slice spiral CT (GE light speed VCT) for head and neck scans were used for all the patients in this study. The patient takes the supine position, establishing a venous access through the median cubital vein, and uses a high-pressure syringe to inject a contrast agent: Ultravist (370 mg/mL) 20 mL at a speed of 4 mL/s. The layer thickness is 0.625 mm and the layer spacing is 0.3 mm. After scanning, the boneless 3DVR reconstructed image is obtained through data processing and image reconstruction. The CTA diagnosis result was determined by two experienced radiologists and a neurosurgeon.

### Statistical Analysis

SPSS 20.0 was used for statistical analysis. The counting data were expressed as constituent ratio and the measurement data as X ± S.T test was used for comparison of measurement data, *χ*^2^ test was used for comparison of counting data and Fisher’s exact test was used for comparison of counting data with expected value less than 1. The parameters with *P* < 0.1 in the univariate analysis were included in the multivariate COX regression analysis model and the test level was α = 0.05.

The nomogram was based on the results of COX regression analysis performed by R version 4.0.2 (The CRAN project, www.r-project.org) and RMS (Version6.0) packages. C-index was used to evaluate the model’s distinctiveness. Bootstrap with 1,000 resamples was drawn to correct the C index. Calibration curves were also drawn to assess consistency between the predicted and actual values. Decision curve analysis (DCA) was used to compare clinical net benefits. *P* value was set below 0.05.

## Results

### Basic Information of Patients

A total of 419 patients were included. Among them, 37 patients including 5 males and 32 females aged 28–73 (53.25 ± 9.61) with the largest diameter of aneurysm (4.94 ± 2.12) mm were enrolled in the observation group. Their intraoperative temporary occlusion rate, temporary occlusion time, the intraoperative aneurysm rupture rate, and the cerebral vasospasm rate is 13.5%, (3.80 ± 0.837) min, 10.8%, 70.3%, respectively. There were 382 patients in the control group, including 132 males and 250 females aged between 30–72 (53.53 ± 9.69), and the maximum diameter of aneurysm was (5.92 ± 2.93) mm, the intraoperative temporary occlusion rate was 10.0%, and the temporary occlusion time was (3.95 ± 1.207) min. The intraoperative aneurysm rupture rate was 1.6% and cerebral vasospasm rate was 27.5%. The baseline data and aneurysm information of patients are shown in Table 1.

**Table 1:** Patient information and univariate analysis of DCI for intracranial aneurysm clipping.

Index	Observation group (*n* = 37)	Control group (*n* = 382)	*t*/*χ*^2^	*P*
Age / years old	53.25 ± 9.61	52.58 ± 9.50	0.347	0.728
Women	32(86.5)	250 (65.4)	6.787	0.009
Hypertension	14 (37.8)	159 (41.6)	0.199	0.655
Diabetes	0 (0)	12 (3.1)	0.334	0.563
Smoking history	5 (13.5)	111 (29.1)	4.071	0.044
Dringking history	5 (13.5)	70 (18.3)	0.531	0.466
Small aneurysms	25 (67.6)	187 (49.0)	4.676	0.031
Uniform shape	34 (91.9)	320 (83.8)	1.698	0.193
Location of aneurysm			2.674	0.6142
ACoA	11 (29.7)	164 (41.1)		
MCA	9 (24.3)	64 (16.8)		
Supraclinoid	13 (35.1)	123 (32.2)		
Paraclinoid	2 (5.4)	13 (3.4)		
Multiple	2 (5.4)	18 (4.7)		
WFNS Classification – Advanced	12 (32.4)	81 (21.2)	2.463	0.117
Hunt and Hess grade			-	0.428
I–III level	36 (97.3)	377 (98.7)		
IV–V level	1 (2.7)	5 (1.3)		
Improved Fisher score			0.480	0.488
I–II level	5 (13.5)	69 (18.1)		
III–IV level	32 (86.5)	313 (81.9)		
Temporary blocking	5 (13.5)	38 (10.0)	0.159	0.690
Operation time /min	213.76 ± 64.30	215.57 ± 75.08	0.142	0.887
Intraoperative aneurysm rupture	4 (10.8)	6 (1.6)	–	0.007
Temporary blocking time /min	3.80 ± 0.837	3.95 ± 1.207	−0.561	0.575
Vasospasm	26 (70.3)	105 (27.5)	28.732	<0.001

### Analysis of Relevant Factors

Univariate analysis showed that women (*P* = 0.009), small aneurysm (*P* = 0.031), intraoperative aneurysm rupture (*P* = 0.007) and cerebral vasospasm (*P* < 0.001) were risk factors for DCI, while smoking history (*P* = 0.044) was a protective factor. When the above factors were included in the multivariate logistic regression analysis model, small aneurysm (*P* = 0.002), intraoperative aneurysm rupture (*P* = 0.004) and cerebral vasospasm (*P* < 0.001) were independent risk factors of postoperative DCI. The results of univariate and multivariate logistic regression analysis are shown in Tables 1, 2, respectively.

**Table 2:** Multivariate Logistic regression analysis of DCI in intracranial aneurysm clipping.

Factors	OR	95% CI	*P*
Gender	0.386	0.102–1.465	0.162
Small aneurysms	3.332	1.563–7.104	0.002
smoking history	1.117	0.102–1.465	0.871
Intraoperative aneurysm rupture	0.122	0.029–0.504	0.004
Cerebral vasospasm	0.153	0.070–0.333	<0.001

### Construction and Validation of the Nomogram

**Figure 1:**
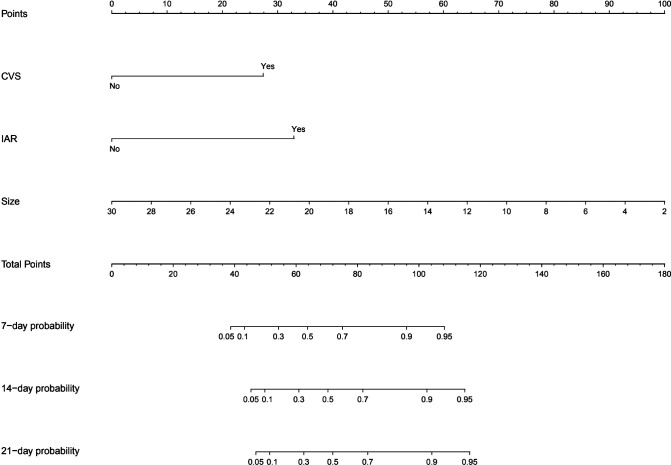
Nomogram for predicting 7-,14-,and 21-day probability of DCI for individual patients.

Based on the multivariate Cox regression results, we selected three independent predictive factors including small aneurysm, intraoperative aneurysm rupture and cerebral vasospasm (Figure 1) to constructed a nomogram in which each of these clinical characteristics was assigned a score on the score scale axis. By adding each individual score and drawing a vertical line between the total score and the survival probability axis, the total score can be easily calculated to determine the 7-,14-, and 21-day probability of DCI for individual patients.

**Figure 2:**
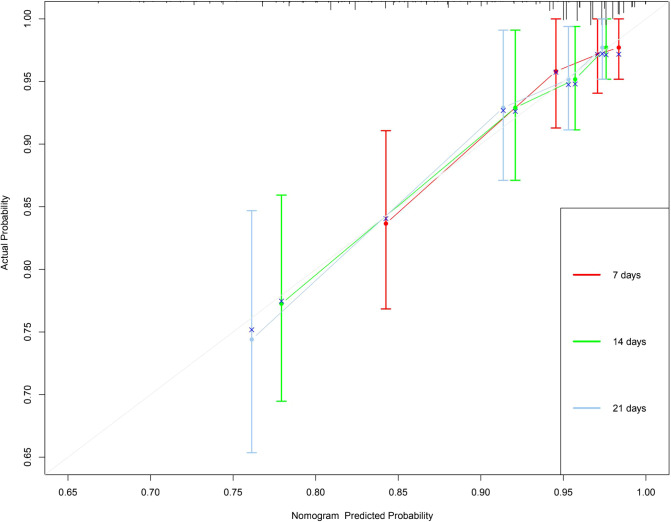
7-, 14-and 21-day calibration curve of nomogram. *X*-axis refers to probability of DCI and the *y*-axis means actual probability. The lines represent the perfect calibration models in which the predicted probabilities are identical to the actual probabilities.

**Figure 3:**
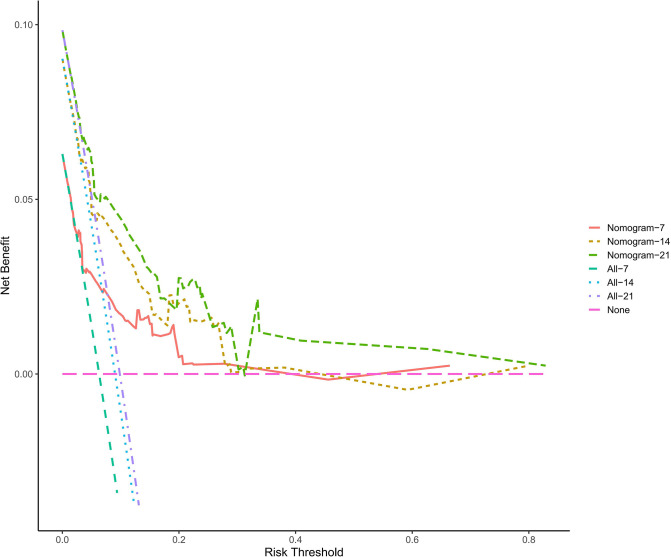
The clinical decision curve for predicting the occurrence of DCI. The *x*-axis represents the threshold probabilities, and the *y*-axis measures the net benefit calculated by adding the true positives and subtracting the false positives.

In this study, the C-index of the nomogram is 0.766 (95% CI, 0.684–0.848), indicating that the prediction accuracy of the model is high. The calibration curve uses 1,000 times of Bootstrap, and the results show that there is a good agreement between the predicted values and the true values of the 7-day, 14-day, and 21-day results in the nomogram prediction (Figure 2). We performed DCA curve comparisons to determine the clinical utility of nomograms. The results show that the nomogram has a good clinical net benefit in predicting the DCI after 7 days, 14 days and 21 days of patients (Figure 3).

## Discussion

In this study, 419 patients were enrolled and three clinical features were selected as prognostic factors in order to constructed a nomogram to predict the incidence of DCI 7-, 14-, and 21-day after intracranial aneurysm clipping. C-index and calibration curve show that the nomogram has good prediction performance. DCA showed that the model had a good clinical net benefit. To our knowledge, our study is the first large-scale retrospective study to develop and validate a nomogram to predict the incidence of DCI after aneurysm clipping.

In this study, 26 patients (70.3%) in the observation group were confirmed to have cerebral vasospasm by CTA or DSA, while this number is only 27.5% in the control group. Univariate analysis suggested that cerebral vasospasm was significantly correlated with postoperative DCI (*P* < 0.001) and further multivariate analysis showed that cerebral vasospasm was an independent risk factor for DCI (*P* < 0.001). Several studies have shown that the probability of DCI induced by cerebral vasospasm after IA surgery is about 11% (7). Cerebral vasospasm is closely related to cerebral infarction and the degree of vasospasm is positively correlated with the occurrence of cerebral infarction (7), which is similar to the results of our work. Ishiguro et al. (8) believed that DCI caused by cerebral vasospasm after subarachnoid hemorrhage may be related to the toxic effect of hemoglobin and calcium channel inactivation caused by other blood components, which, indirectly confirming that cerebral vasospasm is an independent risk factor of DCI. Although all cases in this study were routinely treated with nimodipine after surgery and fasudil was added when cerebral vasospasm occurred, some cases still developed delayed cerebral infarction. In addition, there were still 11 patients (29.7%) in the observation group with no significant cerebral vasospasm in CTA or DSA when DCI occurred in this study. Similar results showed that the area of cerebral infarction in patients may be inconsistent with the range of cerebral vasospasm (9) and some SAH patients had cerebral infarction without manifesting cerebral vasospasm on CT, which suggested that the occurrence of DCI after aneurysm clipping may also be affected by other factors. The failure of several clinical trials of anti-vasospasm drugs also suggested that factors other than cerebral vasospasm contributed to delayed postoperative cerebral infarction (10). These results indicate that the mechanism of DCI after aneurysm clipping is worthy of further study.

Intraoperative aneurysm rupture (IAR) is one of the most critical complications in intracranial aneurysm clipping (11). Aneurysm rupture and bleeding during operation will quickly drown the surgical field so that the difficulty of operation is increased resulting the prolonged parent artery occlusion time, the aggravation of peritumoral vessels disturbance and even excessive or fault clipping. It can also lead to increased subarachnoid hemorrhage and postoperative blood cells disintegrating to release more spasmodic substances (12). The above two factors can significantly increase the occurrence of postoperative cerebral vasospasm and postoperative DCI. Darkwah Oppong et al. (13) reported that the incidence of IAR was about 20%, which confirm that IAR may lead to postoperative cerebral infarction and aneurysm residue and affect the prognosis of patients. In this study, the total incidence of IAR was 2.4%and the incidence of IAR in the observation group and control group were 10.8% (4/37) and 1.6% (6/382), respectively. Univariate analysis showed that the difference between IAR and postoperative DCI was statistically significant (*P* = 0.007). Multivariate analysis showed that IAR was an independent risk factor for DCI (*P* = 0.004). Some researchers believe that the occurrence of IAR is closely related to the clinical experience of the surgeon. The risk of IAR and adverse outcomes after IAR in patients of inexperienced doctors are 1.9 times and 3.21 times of experienced doctors. IAR cases handled by experienced physicians have a better prognosis than those handled by inexperienced physicians (14). In this study, all cases were operated by the same experienced professor and the total incidence of IAR was low. Thanks to the exquisite micro-neurosurgical techniques and experienced surgeons, the accuracy and controllability of aneurysm clipping were greatly increased thus the incidence of delayed postoperative ischemic complications were reduced.

Previous studies have suggested that larger aneurysms are more complex in shape. Meanwhile, tumor neck sclerosis are more obvious and may be accompanied by thrombosis. Repeated adjustment of aneurysm clips during surgery is prone to vascular damage so that ischemic events are easily to happen in large aneurysms and giant aneurysms after surgery (15). PARK et al. (16) also found that large or huge aneurysms in the cerebral artery were prone to cerebral infarction after clipping. The results of our study showed that small aneurysms were more likely to cause delayed cerebral infarction after clipping and small aneurysms (*P* = 0.002) were independent risk factor of postoperative DCI. Previous studies have shown that due to the small size of the tumor, partial vascular wall of the parent artery may be sacrificed when the tumor neck is clipped completely resulting in excessive clipping and leading to lumen stenosis of the parent artery. Endothelial injury caused by over-clipping can also cause vasospasm and even thrombosis resulting in delayed cerebral infarction (17). In addition, the microscopic operation time are prolonged due to the difficulty of clipping of small aneurysm so that the blood vessel of brain withstands long-time vacuum aspiration and thermal radiation, which, may affect the integrity of the endothelial cells and physiological function and also reduce its ability to respond to vasodilation media causing vasospasm and delayed cerebral infarction (17).

Studies have shown that temporary occlusion of the parent artery may be another factor causing the exacerbation of vasospasm after aneurysm clipping (18). Recent studies have confirmed that the temporary occlusion reduces the occurrence of IAR and shorten the whole operation time so that the prognosis of patients is significantly improved. Repeated occlusion of the parent artery within 20 min did not affect the prognostic outcome but multiple repetitions and total temporary occlusion time more than 20 min had a significant impact on the outcome (19). In this study, 5 patients (13.5%) in the observation group received intraoperative temporary occlusion and the temporary occlusion time was (3.80 ± 0.837) min. There were 38 patients (10.0%) in the control group and the temporary blocking time was (3.95 ± 1.207) min. Univariate analysis showed that there was no significant statistical difference of the temporary blocking rate (*P* = 0.690) and the temporary blocking time (*P* = 0.575) between the two groups. There was no correlation between intraoperative temporary occlusion and postoperative DCI and reasonable intraoperative temporary occlusion was safe and beneficial.

Previous studies have found that smoking increases the risk of delayed neurological dysfunction in patients with aneurysm-derived subarachnoid space hemorrhage and smokers are nearly twice as likely to develop delayed neurological deterioration comparing with other patients (20). Different from the above results, univariate analysis in this study showed that smoking history (*P* = 0.044) was a protective factor for DCI after intracranial aneurysm clipping. Some other work has found that smoking is a predictor of good clinical outcomes after aneurysm-derived subarachnoid space hemorrhage because smoking promotes atherosclerosis and inhibits vascular reactivity so that the incidence of vasospasm and DCI could be reduced (21). Although smoking is affected by gender, the multivariate analysis of this study failed to verify its effect on DCI (*P* = 0.871) and this divergence deserves our attention.

## Conclusions

In this study, we found that small aneurysm, intraoperative aneurysm rupture and cerebral vasospasm were risk factors of DCI after clipping. It is suggested that proper treatment of small aneurysm, prevention of aneurysm rupture during operation and active treatment of cerebral vasospasm after operation are keys to reduce postoperative DCI and improve the prognosis of patients. Temporary intraoperative occlusion of the parent artery is safe, beneficial and should not be hesitated if necessary. The effect of smoking on post-clipping DCI is still unclear and needs further study.

Using nomograms to predict the risk of postoperative DCI and determine the clinical treatment is a new concept. Our work is one of the few studies to use nomograms to predict the incidence of DCI after intracranial aneurysm clipping, which includes three comprehensive and easily get variables with good performance. This well-established nomogram is easy to use and exhibits good accuracy and discrimination in predicting results.

### Limitations

This study is a single-center retrospective study that has some limitations. First, there was information bias in the collection process of some data. Some patients with early asymptomatic cerebral infarction may have been wrongly included in the study due to the limitations of the CT scan that could not show acute cerebral infarction. Secondly, most postoperative patients only received CTA instead of DSA after surgery and the above examination was not carried out according to the prospective design so that the reliability is limited. We also need to conduct a multi-center prospective trial with a large number of samples to confirm our conclusions and determine the reliability of the nomogram.

## Data Availability

The original contributions presented in the study are included in the article/Supplementary Material, further inquiries can be directed to the corresponding author/s.
